# A global overview of the most important zoonotic bacteria pathogens transmitted from *Rattus norvegicus* to humans in urban environments

**DOI:** 10.1016/j.imj.2022.07.002

**Published:** 2022-07-30

**Authors:** Sahar Sabour, Taher Azimi, Ahmad Nasser, Nahal Hadi, Amin Mohsenzadeh, Aref Shariati

**Affiliations:** a Department of Microbiology, Faculty of Medicine, Ahvaz Jundishapur University of Medical Sciences, Ahvaz, Iran; b Department of Bacteriology & Virology, School of Medicine, Shiraz University of Medical Science, Shiraz, Iran; c Department of Pathobiology, School of Public Health, Tehran University of Medical Sciences, Tehran, Iran; d Department of Microbiology, Ardabil Branch, Islamic Azad University, Ardabil, Iran; e Molecular and medicine research center, Khomein University of Medical Sciences, Khomein, Iran

**Keywords:** Zoonosis, Bacterial diseases, Zoonotic diseases, Wild rats, Rattus rattus

## Abstract

Zoonotic pathogens, comprising over 61% of all pathogenic microorganisms, can be transmitted from different animals to individuals in numerous ways either in the presence or the absence of a vector. Causing new emerging human infectious diseases, these pathogens could be categorized into 4 groups, bacteria, viruses, parasites, and fungi. Among the wide range of reservoirs for zoonotic pathogens, tremendous attention has been attracted to wild rats*,* due to their global distribution not only in urban environments but also in the sylvatic and agricultural surroundings. For the nonce, zoonotic bacteria transmitted via wild rats have turned into a global public health problem probably due to their ability to induce re-emerging diseases even after eradication and controlling management. Despite the importance of wild rats in spreading pathogens, little data are available about the bacterial diversity present in urban wild rat populations. In this review, we present a complete list of zoonotic bacterial pathogens isolated from wild rats in urban environments.

## 1 Introduction

Zoonotic illness is a general term comprising infections that can be transmitted either in the presence or in the absence of vectors between humans and animals [1,2]. Currently, approximately 61% of 1500 previously diagnosed pathogens can cause zoonotic disease [3] and these pathogens could be categorized into 4 groups, bacteria, viruses, parasites, and fungi [4]. Noteworthy, these zoonotic pathogens could exert an unfavorable impact on tourism, business, and economies [4]. The re-emergence of bacterial zoonotic infections even after eradication or controlling strategies postures these pathogens at the topmost national and international attention and introduce them as a global public health problem [1]. Moreover, the antibiotic-resistant characteristics of zoonotic bacterial infections, which is one of the most important problems in health care systems in developed countries, provide serious clinical challenges for specific patients, especially those with immune deficiency, pregnant status, and young/old patients [5]. There are various pathways by which these bacteria may transmit from infected animals to human hosts. The usual ways of transmission included I) direct fecal-oral route [6]; II) consumption of contaminated food products [7]; III) animal bites and scratches [8]; IV) direct connection with contaminated animals [9]; V) exposing to several vectors including ticks, fleas, lice, and mosquitoes [10]; and VI) exposing to contaminated soil and water resources [11]. Following the first description of the zoonotic illness, multiple reservoirs have been introduced for these pathogens, thus far. In recent years, the global geographic distribution of *Rattus norvegicus* (*R norvegicus*) (Norway rats or brown rat) and *Rattus rattus* (*R rattus*) (black rat, ship rat, roof rat, or house rat) in urban areas, its prolific characteristics as well as its stunning ability to preserve the pathogens, turn this rodent into the main reservoirs for different types of zoonotic microorganisms [12], including *Leptospira* spp., *Rickettsia* spp., *Bartonella* spp., *Coxiella* spp., *Yersinia* spp., and Seoul orthohantavirus [4]. The list of most important zoonotic bacterial pathogens isolated from *R norvegicus* in the different parts of the world is summarized in Table 1. Noteworthy, it has been reported that the reduction in the ecological distribution between reservoirs and humans coupled with the elevation in the density of rodent's population are 2 key factors contributing in the transmission of pathogens to human beings [13,14]. Taken advantages of these facts, it is not surprising that intense interest has currently focused to precisely recognize which rat-associated zoonotic bacterial pathogens are present in urban environments. This review article discussed a complete list of zoonotic bacteria pathogens isolated from wild rats, providing a valuable data for designing and implementing more promising surveillance and intervention policies against this rodent.

**Table 1:** An overview of the most important zoonotic bacterial pathogens isolated from wild rats, worldwide.

**Author**	**Country**	**Published time**	**Pathogens (%)**	**Mode of transmission**	**Samples**	**Methods**
Easterbrook [15]	USA	2007	*L interrogans* (65.3%), *B elizabethae* (34.1%), *R typhi* (7%)	Direct or indirect contact with urine of rodents, hematophagous insects	Serum	ELISA, MAT, IFA
Martin [12]	Germany	2012	*Salmonella* spp. (3.6%)*, Leptospira* spp. (21.3%)*, Yersinia* spp. (1%), *F tularensis* (0%), *C burnetii* (1.3%)	Contaminated water and food of animal origin, contact with the urine of rodents, insects such as ticks and deer flies	Liver and gut	PCR or culture techniques
Chao LL[16]	China	2012	*Borrelia* spp. (42.9%)	Insects such as ticks	Tick specimens collected from Rattus	PCR and sequencing
Sebastian [17]	Germany	2012	*E coli* (77%)	Food and water contaminated with feces	Fecal	Culture technique and biochemical tests
Chelsea [18]	Canada	2014	MRSA (3.5%)	Person-to-person, food, and water contaminated with feces	Oropharyngeal swabs	Culture
Cadhla [4]	USA	2014	*Bartonella* spp. (25%), *C jejuni* (4%), *C difficile* (1%), *C perfringens* (7%), L *interrogans* (12%), EPEC (38), *Y enterocolitica* (1%), *S moniliformis* (17%), *V vulnificus* (0%), *Shigella*/EIEC (5%), *S enterica* (2%)	Animal scratches and bites, contact with the urine of rodents, food, and water contaminated with feces	Fecal, liver, serum, brain, bladder, kidney, urine	PCR
Federico [19]	Brazil	2014	*L interrogans* (63%-83%), *Bartonella* spp. (19%)	Hematophagous insects, animal scratches, and bites, contact with the urine of rodents	Kidney and blood	PCR, immunofluorescence staining
Ayral [20]	France	2014	*L interrogans* (26%)	Contact with the urine of infected animals	Kidney	TaqMan real-time PCR
Chelsea [21]	Canada	2015	*R typhi* (0.4%), *Bartonella* spp. (25.2%)	Hematophagous insects, hematophagous arthropod vectors	Kidney, lung, blood and spleen	Culture, ELISA, PCR
Chelsea [22]	Canada	2015	*E coli*(62.7%), *Salmonella* spp. (0.5%)	Food and water contaminated with feces	Feces	Culture, PCR
Henandez [23]	Mexico	2016	*B burgdorferi* sensu lato (17.2%)	Insects such as ticks	Ear and bladder samples	PCR and sequencing
Elisa [7]	Germany	2017	*Leptospira* spp. (14.3%), *Rickettsia* spp. (0.8%)	Contact with the urine of infected reservoir animals, ticks, fleas	Kidney, ear pinna tissue	PCR, quantitative PCR
Rothenburger [24]	Canada	2017	*B tribocorum* (25.7%)	Hematophagous insects such as fleas, sand flies, lice, cat fleas, ticks, animal scratches, and bites	Blood	PCR
Miriam [25]	Netherland	2018	*Leptospira* spp. (33%-57%)	Contact with the urine of infected reservoir animals	Kidney	Culture, Real-time PCR
Rothenburger [26]	Canada	2018	*C difficile*(12.8%), MRSA (3.7%)	Food and water contaminated with feces	Feces, oral swab	PCR
Nurul [27]	Malaysia	2018	*Leptospira* spp. (16.2%)	Contact with urine of Rattus	Kidney	Culture, PCR
Joshua [28]	Nigeria	2018	*C burnetii* (2.1%), *Rickettsia* spp. (6.3%)	Directly via exposure to their excretions or indirectly via arthropod vectors such as fleas, lice, mites, and ticks	Blood and spleen	PCR
Elena [29]	France	2019	*C burnetii* (8.7%)	Aerosols from animals	Blood	ELISA
Inge [30]	Netherlands	2019	*C difficile* (39.2%)	Food and water contaminated with feces	Gut content	Culture
Azimi [31]	Iran	2021	*E coli* (46%) *Klebsiella* spp. (13%) *Enterobacter* spp. (7%) *Serratia* spp. (2%) *Proteus* spp. (2%) *Shigella* spp. (1%) *Citrobacter* spp. (1%)	Rat urine inhalation of aerosols, contact with rat saliva, eating the contaminated food or water, direct contact by bites of rat fleas, and animal contact	Fecal	Culture, PCR
Azimi [32]	Iran	2021	*Leptospira* spp. (7%)	Direct contact with rodent population or infected livestock and wild animals, contact with surface water, soil, and plants, consumption of contaminated water, direct contact with the urine of infected animals, or contact with a urine-contaminated environment	Serum	ELISA
Azimi [33]	Iran	2021	*S moniliformis* (23%)*, Bartonella* spp. (17%), *C burnetii* (4%)*, Rickettsia* spp. (1%), *L interrogans* (1%), *L kirschneri* (1%)	Contact between human and animal reservoirs, inhalation of aerosols and consumption of contaminated water and food with feces and urine from infected rodents, direct contact by bites of arthropod vectors	Blood and fecal	ELISA, PCR, Real-time PCR
Phirabhat [34]	Thailand	2021	*Bartonella* spp. (38.6%)	Blood-sucking arthropods	Blood	PCR
Le Thi [35]	Vietnam	2021	Bartonella (31.6%), *R typhi* (5.3%), *Rickettsia* spotted fever group (19.5%), *Leptospira* spp. (18%)	Bites of infected ticks, fleas, mites, and lice, cuts in the skin, or drinking contaminated water	Liver and kidney	Real-time PCR
Bruno [36]	Argentina	2022	*Bartonella* spp. (29.4%), *Leptospira* spp. (14.3%)	Bites of infected ticks, fleas, mites, and lice, cuts in the skin, or drinking contaminated water	Kidney	PCR, Real-time PCR

EIEC, enteroinvasive *E. coli*; EPEC, enteropathogenic *E. coli*; IFA, indirect immunofluorescence assay; MAT, microagglutination test; MRSA, methicillin-resistant *Staphylococcus aureus*; PCR, polymerase chain reaction.

## 2 *Bartonella* spp.

*Bartonella* spp. is a gram-negative, rod-shaped, small, slightly curved, fastidious, and facultative intracellular bacteria belonging to the genus of Alphaproteobacteria and the family of the Bartonellaceae [37]. Although *Bartonella henselae, Bartonella quintana,* and *Bartonella bacilliformis* are widely known as potential human pathogens, recent studies have suggested that the genus *Bartonella* is comprised of 33 species, more than fifty percent of them could be harbored by rodents [38], [39], [40]. Among these species, 6 were identified as zoonotic pathogens, which are *Bartonella vinsonii* subsp *Arupensis, Bartonella grahamii, Bartonella washoensis, Bartonella tribocorum, Bartonella elizabethae*, and *Bartonella alsatica* [41], [42], [43]. The prevalence rate of *Bartonella* infection in rodents such as rats has been surveyed in several studies, which varied from 8.7% to 25%[19,21]. These studies also revealed that the prevalence of *Bartonella* infection as ectoparasites of rats is varied from 3.5% to 57.1% [4,19,21]. *Bartonella* genus could effectively transmit to a wide range of hosts through several mechanisms. The great frequency of the *Bartonella* genus in mammalian reservoirs, inducing long-term bacteremia in hosts, and last but not least the ability of this genus to adapt to definite vectors are suggested to be the main mechanisms that *Bartonella* could exploit to widely transmit to different hosts [40,44,45]. *Bartonella* spp., especially *B elizabethae* complex sensu lato, is usually transmitted between rodents (rats) and humans by hematophagous insects such as fleas, sand flies, lice, cat fleas, and possibly ticks or via animal scratches and bites [40,46]. Recent research reported that fleas play an important role in the *Bartonella* cycle, due to harbor a high diversity of *Bartonella* spp. and strains and showed great efficiency in the transmission of these bacteria among rodents [45,47]. The clinical manifestations of *Bartonella* zoonotic species are ranging from mild symptoms, including flu-like illness, fatigue, muscle, and joint pain, to more severe manifestations, such as myocarditis, endocarditis and neurological signs, arthritis, hepatitis, and arthralgia, which are present especially in immunosuppressed patients [48,49]. For the nonce and among the growing list of laboratory diagnostic techniques used for the detection of *Bartonella*, it seems that isolation of the organisms by specifying microbiological cultures, performing the molecular assays such PCR, serological assays, and immunohistochemistry (IHC) is the most beneficial approaches [49], [50], [51]. Noteworthy, the “gold standard” method for diagnosis of *Bartonella* infections is specific microbiological culture techniques including lysis centrifugation, using enriched culture media such as insect biochemical composition growth media and cell culture isolation [37]. While a considerable number of efforts are still focused on identifying a beneficial treatment strategy for *Bartonella* infection, recent investigations have shown that several antibiotics such as aminoglycosides, fluoroquinolones, penicillin, cephalosporins, chloramphenicol, and rifampin seems to be effective against *Bartonella* genus. Nevertheless, several species of *Bartonella* genus, particularly *B bacilliformis,* are resistant to several antibiotics such as quinolones and macrolides [52].

## 3 *Borrelia* spp.

The *Borrelia* genus is compromised from several different species of spirochetes and is responsible for different types of diseases in their hosts [53]. Lyme disease (LD) is an important emerging tick-borne zoonosis infectious disease that is primarily caused by *Borrelia* species. *Borrelia burgdorferi* sensu stricto is the main cause of LD and is comprised of twenty genospecies that are distributed in different geographical regions [54]. Among twenty genospecies, 5 genospecies including *Borrelia garinii, Borrelia afzelii, B burgdorferi, Borrelia bavariensis*, and *Borrelia spielmanii* are considered human pathogens [55]. Additionally, 3 genospecies including *Borrelia lusitaniae, Borrelia valaisiana*, and *Borrelia finlandensis* are suspected to be human pathogens and it has been suggested that *Borrelia bissettii* could not exert any clinical symptoms in human beings [53]. Based on the reports from the Centers for Disease Control and Prevention (CDC), it is well-established that while *B burgdorferi* is the main pathogen responsible for LD in the United States, *B afzelii* and *B garinii* induce LD in Europeans and Asians. Despite the tremendous improvements in the management and treatment strategies over the past decades, LD remains a main public health concern, accounting for approximately 30,000 new cases worldwide [56], [57], [58], [59]. Generally, the *Borrelia* genus is acquired from rodent reservoirs, such as mice and rats, and spread among different hosts by hard-bodied ticks of the genus *Ixodes* [60]*.* The prevalence rate of *Borrelia* spirochetes in Ixodes granulatus ticks collected from Rattus losea on Kinmen Island of Taiwan, China has been surveyed in a study, which is varied in nymph, male, and female stages of Ixodes granulatus ticks with an infection rate of 42.9%, 36%, and 52.7%, respectively [16]. Moreover, in another study, the prevalence rate of *B burgdorferi* sensu lato in rodent reservoirs has been surveyed, which is 17.2% [23]. In rodent hosts, the *Borrelia* genus exploits several immune evasion strategies, leading to long-term infectious disease [61]. These resuscitative strategies are as follows: I) antigenic variation in outer surface protein [62], II) extracellular matrix degradation leading to inappropriate macrophage activation [63], III) spirochete shape, allowing *B burgdorferi* to diffuse and persist [64], IV) complement inhibition [65], V) *Borrelia* adhesins that interact with different host tissues and contribute to diffusion and persistence [66], VI) Tick salivary proteins that suppress pro-inflammatory responses in the host [67], VII) host-pathogen coevolution that contributes to host specialization, leading to the evolution of virulence- and infectivity-associated genes [68], and VIII) adaptive immune response interference [69]. LD could affect different organs, including skin, musculoskeletal, nervous system, cardiovascular system, ocular system, hepatobiliary system, respiratory system, renal system or urinary tract, and hemato-lymphoid system [70]. In a general term, clinical manifestations of LD could be divided into 2 main stages, early stage of infection and late stage of infection. Of note, the early stage of infection itself is divided into 2 stages: localized infection (stage I) and disseminated infection (stage II). The late stage of infection often leads to persistent infections (stage III) [71]. The important clinical manifestation of stage I is erythema migrans, however, other manifestations have also been reported for this stage of disease. These symptoms include secondary annular lesions, malar rash, diffuse erythema or urticaria, lymphocytoma, migratory pain in joints, muscles, tendons, bursae, bones, brief arthritis episodes, myositis, osteomyelitis, panniculitis,meningitis, facial palsy, radiculoneuritis, mononeuritis multiplex, myelitis, mild encephalitis, cerebellar ataxia, atrioventricular nodal block, myopericarditis, pancarditis, conjunctivitis, iritis, choroiditis, panophthalmitis, retinal hemorrhage or detachment, mild or recurrent hepatitis, dry cough, sore throat, microscopic proteinuria and/or hematuria, orchitis, lymphadenopathy, severe malaise and fatigue [58,72,73]. In addition, acrodermatitis chronica atrophicans, localized scleroderma-like lesions, prolonged arthritis episodes, chronic arthritis, periostitis, joint subluxations, enthesopathy, chronic encephalomyelitis, spastic paraparesis, ataxis gait, chronic axonic polyradiculopathy, generalized/regional lymphadenopathy, and keratitis are the main clinical signs associated with stage III [71]. The definitive guidelines for the diagnosis of LD is not available and also the therapeutic approach for *Borrelia* infection is still an open to debate. However, based on different stages of the disease, various class of antibiotics are prescribed. For example, according to the CDC guideline, several antibiotics including cefotaxime, ceftriaxone, doxycycline, and penicillin G are effective in LD-associated neuroborreliosis [56,74]. Currently, according to the National Institute for Health and Care Excellence (NICE) guideline, the laboratory diagnostic technique for the detection of *Borrelia* infections, especially LD is as follows; I) culture (not performed routinely), II) PCR-based analysis, III) serological assay (performed routinely) including enzyme immunoassay, ELISA, and IV) confirmatory test, such as immunoblots. Moreover, it is suggested that due to the false-positive results of sociological methods, positive results must be sent to reference laboratories for confirmation [58,75,76].

## 4 *Yersinia* spp.

The genus of *Yersinia,* a gram-negative, non-spore-forming coccobacilli, could grow under various atmospheric conditions including aerobic and anaerobic culture conditions and they could survive in a wide range of temperatures (between 0 °C and 45 °C) [77]. This genus of bacteria consists of 18 species, however, only 3 species of them, including *Yersinia pestis, Yersinia enterocolitica*, and *Yersinia pseudotuberculosis,* are pathogenic for either humans or animals [78,79]. The name *Y pestis* brings plague, the most deadly disease worldwide, to mind, which is transmitted between the wildlife rodents and humans by fleas, inhalation of respiratory aerosols, and close contact with infected tissues [80]. For the nonce, the evidence of several pandemics of the plague from different parts of the world revealed that probably this disease first originated from central Asia, and subsequently spread throughout Africa and Europe [81,82]. Another pathogenic bacteria in this genus is *Y enterocolitica,* an opportunistic pathogen transmitted to humans from different sources, including raw meat, milk and milk products, vegetables, bean sprouts, poultry, eggs, seafood, and stewed mushroom [83]. The prevalence rate of *Yersinia* spp. in rodents such as rats has been surveyed in several studies, which is varied from 1% to 25% [4,12]. These bacteria are mainly a gastrointestinal pathogen that usually causes self-limiting food-borne enteric illness (Yersiniosis); however, several abnormalities include terminal ileitis, mimicking appendicitis, and erythema nodosum, glomerulonephritis, mesenteric lymphadenitis, arthritis, and septicemia in immunocompromised individuals reported after *Y enterocolitica* infections [84,85]. *Y enterocolitica* has different biotypes (6 biotypes included 1A, 1B, 2–5) and numerous serogroups (more than 70 serogroups). The serogroups O: 3, O: 5, 27, O: 8, and O: 9 are predominantly human pathogens [77]. Moreover, it is well-established that biotype 1A is non-pathogenic, biotypes 2–5 are weakly pathogenic, and biotype 1B is highly pathogenic [86]. The virulence factors for *Y enterocolitica* have been enumerated in several studies and are summarized as follows: I) mucoid *Yersiniae* factor (MyfA); II) *Yersinia* adhesin (YadA); III) invasin (InvA); IV) attachment-invasion locus (Ail) protein; V) *Yersinia* outer membrane proteins (Yops); VI) *Yersinia*-stable toxin type I A (YstIA); VII) *Yersinia*-stable toxin type I B (YstIB); VIII) *Yersinia*-stable toxin type I C (YstIC); IX) transcriptional activator of the *Yersinia*; X) *Yersinia* modulator (YmoA); XI) Flagella; XII) Lipopolysaccharide (LPS); XIII) Ysc T3SS and Ysa T3SS; and XIV) High-Pathogenicity Island (HPI) [79,80,85,87]. Given the widespread of *Yersinia* infections, multiple laboratory diagnostic techniques have been applied to more precisely detect these bacteria. These techniques comprise immunoassays, including ELISA,  immunomagnetic separation polymerase chain reaction (IMS-PCR), Latex agglutination, cultural methods in selective enrichment media (PBS/ITC/TSB/BOS), and selective differential plating (SSDC/CIN), and molecular-based methods, such as colony hybridization, microarray, and PCR [84,88]. Discovery of the effective therapeutic approaches for *Yersinia* infections has progressed at an astonishing pace and ushered into the new field of therapies. Although tetracycline, chloramphenicol, gentamicin, and cotrimoxazole are the most recommended antibiotics by the World Health Organization (WHO) for the treatment of *Yersinia* infections, the results of the recent investigations have declared that third-generation cephalosporins and fluoroquinolones are the better alternatives in the treatment strategies against *Yersinia* infection [78,85,89].

## 5 Streptobacillus moniliformis

*Streptobacillus moniliformis* (*S moniliformis)* and *Spirillum minus* (*S minus*) are non-motile, highly pleomorphic, and non-acid-fast facultative anaerobic gram-negative bacterium that causes 2 important zoonosis illnesses including Rat-bite fever (RBF) and Haverhill fever [90]. It has been shown that *S moniliformis* could colonize in the oronasopharynx or nasopharynx of different rodents, especially Rattus. Moreover, it has been indicated that Rattus shed the bacterium through its saliva and urine [91]. The other ways by which this organism can be transmitted to its vulnerable hosts are mediated either through the bite and scratches of Rattus or consumption of contaminated foods [92,93]. Of note, the person-to-person transmission has not been reported, thus far [92]. The prevalence rate of *S moniliformis* infection in rodents such as rats has been surveyed in a study, which is 17% [4]. Although the virulence factors of *S moniliformis* are not completely well-defined, several bacterium properties, such as α hemolysin, DNase, LPS, and adhesins have been enumerated in recent studies as plausible virulence factors for this bacteria [91]. The clinical manifestation of RBF and Haverhill fever are similar to each other and includes high fever, chills, headache, nausea and vomiting, characteristic skin eruptions, arthralgia, and myalgias [94,95]. Moreover, several complications such as metastatic abscesses, pneumonia, myocarditis, pericarditis, endocarditis, meningitis, and amnionitis have been reported to be associated with *S moniliformis* infections [90]. The laboratory diagnostic methods widely used for the detection and isolation of *Streptobacillus* infection are as follows: I) bacterial cultivation from clinical samples including blood, synovial, pus, or other fluids on blood agar or tryptose soy agar enriched with 10%–20% rabbit serum [92,96]; II) hemagglutination [90]; III) serum agglutination [97]; IV) mass spectrometry; V) Fourier transform-infrared spectroscopy (FT-IR); VI) PCR; VII) immunoassays-based analysis including ELISA, complement fixation test, and indirect immunofluorescence assay (IFA); and VIII) SDS-PAGE [91,98]. Although *S moniliformis* is remarkably sensitive to a wide range of antibiotics, such as cephalosporins, carbapenems, penicillins, erythromycin, tetracycline, teicoplanin, clindamycin, and vancomycin; this bacteria still remains one of the leading causes of person-years of life lost in untreated cases, with the mortality rate of 7% to 53% [90,92,99].

## 6 *Shigella* spp.

*Shigella* spp., a gram-negative, anaerobic, and rod shape enteropathogen, are belonged to the Enterobacteriaceae family. These bacteria generally contribute to gastrointestinal abnormalities (shigellosis) by invading the colonic epithelium in humans and other primates [100]. It is worth mentioning that this bacteria is considered a public health concern all around the world [101]. Based on CDC and Food-borne Diseases Active Surveillance Network (FoodNet) reports, *Shigella* was considered the third most detected bacterial pathogen in many types of foods, such as fruits and vegetables, and one of the most important food-borne pathogens [100,101]. The bacteria belonging to the *Shigella* genus are antigenically various and based on serologic or biochemical reactions separated into 4 serogroups or species, including I) serogroup A (*Shigella dysenteriae*); II) serogroup B (*S. flexneri*); III) serogroup C (*Shigella boydii*); IV) serogroup D (*Shigella sonnei*) [102]. Several severe abnormalities such as mucosal ulceration [103], colonic inflammation, and disruption in intestinal barrier function have been reported to be associated with this infection [104]. Epidemiologic reports have reported that approximately 164.7 million new cases of shigellosis are diagnosed each year, and among them, 1.1 million individuals lose their lives due to this infection [105]. The geographical distribution of *Shigella* spp. is varied; for instance, *S boydii* is predominantly detected in Central and South America, whereas *S flexneri* and *S sonnei* are mainly observed in areas of endemic infection and the developed countries, respectively [102,106,107]. Among *Shigella* species, *S dysenteriae* serotype 1 has the highest fatality rate, with the mortality rate varying from 5 to 15% [105]. Several ways have been identified for *Shigella* spp. transmission, such as I) fecal-oral route; II) person-to-person contact (due to low infectious dose; 10 to 100 organisms); III) ingestion of feces contaminated food or water; IV) transmission by contaminated swimming pools and flies [101,102,108]. Several studies have surveyed the presence of *Shigella* spp. in Rattus, however, a study has revealed that the prevalence of this bacteria is about 5% of the fecal samples collected from Rattus, suggesting that Rattus could probably serve as a carrier of the *Shigella* genus [4]. *Shigella* spp. has various effectors, including OspB, OspE1, OspE2, OspZ, OspI, OspG, OspF, OspC3, IpgB1, IpgB2, IpgD, IpaA, IpaB, IpaC, IpaD, IpaJ, IpaH, IcsB, and VirA [103]. By targeting the colonic epithelium in humans, *Shigella* spp., in particular *S dysenteriae,* predominantly cause major complications for individuals, especially for infants, children, and immunocompromised patients [100,105]. The clinical manifestation of shigellosis is various and included fever, headache, malaise, anorexia, vomiting, mild watery diarrhea, bloody mucoid diarrhea, hemolytic-uremic syndrome, renal failure, and painful abdominal cramps, post reactive arthritis, and chronic arthritis of the joints [109]. The “gold standard” method for the diagnosis of *Shigella* infection is conventional microbiological culture techniques. Noteworthy, stool culture is a cornerstone in the diagnostic approaches [100,110]. The maintenance of hydration and electrolyte balance is the most effective strategy to cure shigellosis [102]. According to the WHO guideline, the main antibiotics used for shigellosis treatment are ciprofloxacin, and 3 second-line antibiotics are cefixime, azithromycin, and ceftriaxone [111].

## 7 *Campylobacter* spp.

The genus *Campylobacter* contains a set of fastidious spiral-shaped rod bacteria that cannot produce spores. The motility pattern of this gram-negative bacteria resembles a corkscrew that is mediated through 1 or 2 polar flagella present on 1 or both ends of the cell. Given its microaerophilic feature, this bacterial family requires 5% O_2_, 10% CO_2,_ and 85% N_2_ at a narrow range of temperatures ranging from 30 to 46 °C [112], [113], [114]. The genus *Campylobacter* contains 26 species, among them; *Campylobacter jejuni* and *Campylobacter coli* are commonly known to cause gastroenteritis in humans. *Campylobacter concisus*, a nonzoonotic *Campylobacter* species, has a tight association with periodontitis and gingivitis. Moreover, other family members, such as *Campylobacter upsaliensis, Campylobacter ureolyticus, Campylobacter hyointestinalis,* and *Campylobacter sputorum* can cause periodontitis and gastrointestinal disorders [115,116]. Since *Campylobacter* is a normal flora of the gastrointestinal tract of birds, pigs, cattle, dogs, and cats, this bacterium is mostly transmitted to humans through animals. *Campylobacter* is transmitted by the consumption of milk, meat, fruits, and vegetables; however, this bacterium mainly infected human being through the consumption of poultry meat [108,109]. A study conducted in the Netherlands showed the isolation of *Campylobacter* spp. from the house rats and wild brown rats in organic farms [117]. Furthermore, another study showed that the rats which were collected from 44 locations across the island of Trinidad could be the source of *Campylobacter* spp., with an isolation frequency of 3.4%. It is noteworthy that this study indicates the possibility of human food and environmental contamination since the majority of the rats were trapped in the areas close to the human habitation and markets. It should be noted that the prevalence of this bacterium was lower in Trinidad compared to those of France and Portugal with the prevalence of 21.8% and 57.4% respectively [118,119]. Meerburg reported that *Campylobacter* spp. is present in the house mice and brown rats. So, effective on-farm rodent management is recommended to prevent the transmission of these bacteria to livestock and human [117]. *Campylobacter* species (except *Campylobacter gracilis*) can move and adhere to the epithelium by various factors such as CadF (Campylobacter adherence Factor) and CapA (Campylobacter protein A), which in turn can destroy the host cells by producing various toxins. Cytolethal Distending Toxin (CDT) is one of the most widely known toxins that induce cell distension and can cause cell death by fragmenting the nucleus [120,121]. The range of diseases caused by *Campylobacter* can vary from self-limiting diarrhea to severe dysentery with inflammation. The results obtained from an increasing number of studies have suggested that the nature of the disease is usually more severe in industrialized countries as compared with developing countries [122]. In addition to the gastrointestinal and domestically associated food-borne infections, several systemic diseases, such as Guillain-Barre Syndrome (GBS), Miller Fisher Syndrome (MFS), and *Campylobacter*-related arthritis, have been reported to have a tight association with *Campylobacter*. Moreover, it is revealed that infection with *Campylobacter* can increase the risk of Inflammatory Bowel Disease (IBD) and Irritable Bowel Syndrome (IBS) [123], [124], [125]. For the nonce, the utilization of the special culture media that contain various antibiotics to suppress the growth of other bacteria is the most appropriate technique to detect *Campylobacter*. For example, Butzler is one of *the Campylobacter* culture media that contains bacitracin, novobiocin, cephalothin, and colistin to suppress other bacteria. Moreover, Blaser and Skirrow, which both contain polymixin and vancomycin, are also used to isolate *Campylobacter* [113,126]. In the era of novel technologies and experimental methods, other state-of-the-art techniques, such as MALDI-TOF MS (Matrix-Assisted Laser Desorption Ionization-Time of Flight Mass Spectrometry), nucleic acid amplification tests (NAATs) and Stool Antigen Assays are employed to more precisely identify various genera of *Campylobacter* [127]. The widespread use of antibiotics in livestock and poultry farming leads to the development of antibiotic-resistant phenotypes in the *Campylobacter* genus [128]. Recent studies have shown that *Campylobacter* spp. isolated from wild rats are highly resistant to cephalothin, trimethoprim/sulfamethoxazole, streptomycin, ampicillin, and nalidixic acid [119,129]. Although endorsed attempts have been made to use vaccination against *Campylobacter* in humans and livestock, the best way to control bacterial infections is to improve health infrastructure and rodent control strategies in urban communities and farms [117,130].

## 8 *Coxiella burnetii*

*Coxiella burnetii* (*C burnetii*) is a small obligate intracellular coccobacillus that is categorized as a gram-negative bacterium due to the presence of lipopolysaccharide (LPS) in its membrane [131]. This microorganism possesses 2 different forms throughout its life cycle; a large-cell variant (LCV) or replicating the form and a small-cell variant (SCV) or non-replicating form, which the latest protects *Coxiella* against extreme environmental conditions, such as heat, pressure, and ultraviolet inactivation [132]. *C burnetii*, a potent pathogen of pets and domestic ruminants, is responsible for inducing asymptomatic infection, leading to abortion and parturition, especially in sheep and goats [131,133]. It has been reported that inhalation of aerosol from infected animals containing even a small proportion of bacteria (1–10 bacteria) could be pathogenic for human beings. Apart from inhalation, it seems that ticks can also be involved in the transmission of this pathogen from animals to humans [134]. A previous study conducted in France has reported that *C burnetii* has widely distributed in *R rattus* and *Mus musculus* domesticus. The overall seroprevalence of anti-*C burnetii* was 9.32% from *R rattus* and 13.33% from *Mmusculus* domesticus [135]. Further study in Egypt which was performed on 75 rats (55 *R norvegicus* and 20 *R rattus*), showed that the overall prevalence of *C burnetii* in the feces of investigated rats was 6.7%, moreover, the prevalence of *C burnetii* was higher among the *R rattus* (15%) in comparison to the *R norvegicus* (3.6%)[136]. A study conducted in Nigeria in a peri‑urban setting showed that 2.5% of *R norvegicus* and 2.1% of *R rattus* were positive for *C burnetii* [28]*.* Fronda et al. reported a wide distribution of *C burnetii* in north-western African islands, in both *R rattus* and *Mmusculus* rodents. Besides, they suggested that controlling these small rodents could prevent Q fever, since *C burnetii* can infect a large range of hosts, including livestock and human [137]. The results of a study conducted in the Netherlands also showed the isolation of *C burnetii* DNA from 4.9% of the *R norvegicus* and 3.0% of the *R rattus* spleen. The investigations have reported the presence of *C burnetii* infection in the kidney, lung, liver, and intestinal tissues but this infection was not evident in the heart, brain and pancreas tissues [138].

So, *R norvegicus* as a commensal nature play an important role in the persistent dissemination of endemically circulating *C burnetii* in environments to human and domestic. Akin to this is the predatory manner of cats on these rats which are typically related to the high prevalence of *C burnetii* so, establishing a sylvatic cycle between hosts, reservoirs, and vectors [28]. High bacterial stability in the environment and the unique ability to disperse via aerosols give *C burnetii* a pathogenic property, classifying this bacterium as one of the dangerous pathogens. The most common disease caused by *C burnetii* in humans is Q fever which is asymptomatic or self-limiting in a wide range of patients (60%). The acute form of the disease is associated with fever, pneumonia, hepatitis, and endocarditis, which can be very dangerous and deadly in patients with underlying illnesses [139,140]. Since the wide variety of animals could be regarded as a reservoir for *C burnetii,* this bacterium could conveniently transmit to humans from pets and livestock. Although there are conflicting reports about the human-to-human transmission of *C burnetii*, some evidence has suggested that blood transfusion, sexual transmission, and an infected pregnant woman could be considered as other forms of bacterial transmission threatening human beings [141,142]. As it was mentioned earlier, it has been suggested that ticks can also be one of the carriers of *C burnetii* and transfer this bacterium from animals to humans [143]. Since *C burnetii* is transmitted by aerosol, this bacterium could be served as a bioterrorism tool. Given this, it is not surprising that tremendous efforts have been devoted to rapidly and precisely identifying this bacterium. With the advent of Real-time PCR-based analysis in the diagnostic approaches, this technique has opened a new gate into *C burnetii* detection, since it can be used for not only environmental samples but also for ticks and various human specimens. For Real-time PCR, whole blood or buffy coat aliquots collected in Ethylenediaminetetraacetic acid (EDTA) or citrate at onset of symptoms and before antibiotic treatment; however, serum, urine and throat swab can also be used for disease diagnosis [144]. Given to be the time-consuming, difficult, and safety profiles, cultivation-based methods are not recommended for the diagnosis of this bacteria [145]. Even though *C burnetii* is resistant to a wide range of antibiotics, it has been reported that doxycycline, either alone or in combination with fluoroquinolones could exert a favorable impact on the treatment strategies of this bacteria [146]. As mentioned above, small rodents can play a very important role in transmitting *C burnetii* to humans and domestic animals. Therefore, the wild rats also should be included in the planning for the prevention of Q fever disease.

## 9 *Escherichia coli*

*Escherichiacoli* (*E coli*), as a member of the family Enterobacteriaceae, exists as normal flora in the digestive tract of humans and animals. A variety of diseases can be stimulated by this bacterial family, such as urinary tract infection (UTI), sepsis, gastrointestinal tract infections, hemolytic-uremic syndrome (HUS), and meningitis [147,148]. Diarrhea is one of the most important diseases caused by *E coli*, which leads to the deaths of thousands of people around the world (300,000–500,000 deaths per year), especially children (760,000 children under 5 years old) [149]. Based on antigenic differences, virulence factors, site of colonization, patterns of bacterial attachment to host cells, and production of toxins, 6 major *E coli* pathotypes could cause diarrhea: Enteroaggregative *E coli* (EAEC), Shiga-toxin-producing *E coli* (STEC) (Verocytotoxin-producing *E coli* (VTEC) or Enterohemorrhagic *E coli* (EHEC), Enteroinvasive *E coli* (EIEC), Enteropathogenic *E coli* (EPEC), Enterotoxigenic *E coli* (ETEC), and Diffusely adherent *E coli* (DAEC) [150]. EPEC, being highly prevalent in hospitals, is the most important pathogen that causes persistent diarrhea in children [151]. Noteworthy, watery diarrhea with abdominal pain and fever is one of the most common symptoms of diarrhea caused by EPEC [152]. The common features and virulence factors of EPEC isolate include bundle-forming pilus (BFP), biofilm formation, Intimin, and lymphocyte inhibitory factor [149,153]. Given the existence of the *stx* gene, a gene encoded Shiga toxin, it seems that STEC-associated diarrhea is similar to diarrhea caused by *Shigella* spp. It has been reported that this type of diarrhea could be transmitted through fecal contaminated hands and foods. The symptoms of this infection in humans are hemorrhagic colitis, fever, and HUS [154]. EIEC mostly targets colon epithelial cells, M cells, and macrophages, causing infections ranging from mild watery diarrhea to severe inflammatory bacillary dysentery (shigellosis) which is coupled with strong abdominal cramps, fever, chills, and stools containing blood and mucus [155]. EAEC is one of the main causes of diarrhea in travelers and immunocompromised children in developing countries [156]. ETEC is a major cause of traveler's diarrhea in developing countries. It has been demonstrated that the incidence of this bacteria is typically rare in advanced countries; however, some surveys are reporting its outbreaks in some areas of these countries [157,158]. The results of a study conducted by Nkogwe et al. showed that 83.3% of the rats collected from various locations across Trinidad and Tobago were positive for *E coli,* and all of the isolates were negative for the O157 strain [129]. Another study in Canada reported that *E coli* was isolated from 62.7% (397/633) of the urban rats (*R norvegicus* and *R rattus*), noteworthy, 4.3% of these bacteria showed multidrug-resistant properties. This study also showed that *R norvegicus* had a greater possibility of carrying *E coli* compared to the *R rattus*, and there was geographic clustering of specific resistance patterns and STEC serotypes [159]. Once *E coli* enters the digestive system, the bacteria immediately attach and colonize the intestinal cells, which allows it to not only evade from the host immune system but also to attack host cells by producing toxins [149]. Antibiotics can reduce the symptoms and the duration of the disease, but some *E coli* species displayed resistance to antibiotics as the result of the overuse of antibiotics in the past 50 years. Furthermore, recent studies have shown that *E coli* species isolated from rats have high resistance to tetracycline, chloramphenicol, sulphamethoxazole, streptomycin, and ampicillin [129,160]. A study which was conducted in Hong Kong reported that 7.7% and 13.9% of the samples isolated from *R rattus* and *R norvegicus*, respectively, had ESBL-producing *E coli*[161]*.* It should be noted that according to the geographical locations, the prevalence of ESBL carriage among *R norvegicus* was significantly different [162]. Another investigation in Germany reported the possibility of the role of the urban rats in transmitting multiresistant and virulent *E coli* strains[17]. This strain showed a high rate of in-vivo pathogenicity using a chicken infection model [17]. Moreover, as a threat to public health, rats in different regions of the world are potential carriers for drug-resistant *E coli* [129,163]. According to the studies conducted in different countries on the isolation of *E coli* strain from rats and other wildlife, it is obvious that rats can transmit this important pathogen to other animals and cause foods and the environment contaminations. Therefore, it is critical to improve environmental sanitation by using bacteriophage, vaccines and bacteriosins to inactivated/eliminate pathogenic *E coli* from food supplies [149,162].

## 10 *Francisella tularensis*

*Francisella tularensis* (*F tularensis*) is an intracellular gram-negative bacterium that can infect various types of eukaryotic cells, including dendritic cells, neutrophils, and alveolar type II epithelial cells [163]. *F tularensis* is divided into 4 clinically relevant subspecies: *tularensis* (type A strains), *holarctica* (type B strains), *mediasiatica,* and *novicida* [164]. *F tularensis* can infect a high spectrum of mammalian and arthropod cells [165]. Once entering into the macrophage, the bacterium escapes from the phagosome and enters into the cytoplasm, where it can replicate repeatedly, and thereby, trigger apoptotic pathways in the host cells. Investigating the pathogenesis of the bacteria in animal models revealed that probably *F tularensis* infects phagocytic cells as the primary step and then, before inducing any severe immune reaction, migrates from these cells to various organs, such as the liver, spleen, and lungs [166,167]. Noteworthy, it is well-established that *F tularensis* does not produce toxin and destroys the host cell through direct invasion [165,168]. The most important disease caused by *F tularensis* is tularemia, with high prevalence rate in parts of the North America, Eurasia and Northern Hemisphere [169]. Tularemia could also be observed in various animals, in particular lagomorphs and small rodents that could effectively transmit bacteria to humans. This finding suggests the significant role of voles, rats, mice, lemmings, and beavers in spreading this disease to human beings [164,170]. As a very acute disease, Tularemia in the house mouse and *Mmusculus* is usually accompanied with development of the following symptoms: sepsis plus spleen and liver enlargement [164]. Moreover, it has been reported that arthropods can be contaminated with *F tularensis*, passing these bacteria to humans*.* The entry of bacteria through the eyes, skin, digestive system, and respiratory tract leads to the formation of oculoglandular, ulceroglandular, oropharyngeal tularemia, and pneumonic tularemia forms, respectively [164,171,172]. It should be noted that pneumonic and typhoidal tularemia are the most dangerous forms of disease and, if left untreated, could be responsible for the death of 30%–60% of patients [173]. To provide a better landscape for the diagnosis of tularemia around the world, patients' clinical symptoms, epidemiological information, and serological tests should be used. Among the serological tests used for the diagnosis of tularemia, micro agglutination test and immune fluorescence assay are the most important ones. Moreover, ELISA-type and western blot assays could be useful in the detection of these bacteria [174,175]. Due to the intracellular property of *F tularensis* and given the poor penetration of beta-lactams into the infected cells, it seems that ciprofloxacin, levofloxacin, doxycycline, and gentamicin are currently the better choices for the treatment of this bacteria [176,177]. It is worth mentioning that various tularemia vaccines, such as the killed “Foshay” vaccine, subunit vaccines comprising *F tularensis* protein(s) or lipoproteins(s) in an adjuvant formulation, and the *F tularensis* Live Vaccine Strain (LVS) are now under intense investigations [178].

## 11 *Leptospira* spp.

According to 16SrRNA, the *Leptospira* genus is divided into saprophytes, intermediate pathogenic, and pathogenic species. Among different species, *Leptospirainterrogans, Leptospiranoguchii*, and *Leptospiraalstoni* are pathogenic and could induce severe and deadly diseases in humans [179,180]. The genome of this family is larger than that of other spirochetes, which seems to be the underlying reason supporting the ability of *Leptospira* spp. to live in different hosts and environments [181,182]. The main disease which is associated with the *Leptospira* genus is leptospirosis. This disease occurs in all parts of the world and especially in tropical and subtropical regions. The incidence of this bacteria has been widely reported in America, Africa, and Asia [183]. *L interrogans* is colonized in the proximal renal tubules of the animal hosts (both wild and domestics animal) and then spread through the urine into the environment [184].

Indeed, rodents commonly infected by *Leptospira* spp., keep bacteria as a chronic infection in the renal tubules and excrete them in their urine throughout their life span, generally in high amounts [185]. Therefore, humans can get leptospirosis through direct or indirect exposure to urine or urine-infected equipment and water. This disease can be manifested either as a mild self-limiting illness or as a severe disease (febrile illness to jaundice, pulmonary distress, acute renal and hepatic failure, and potentially lethal pulmonary hemorrhage) that can cause mortality if left untreated. The motility is one of the main characteristics of the pathogenicity of *L interrogans*. Since this bacteria could easily pass through the viscous media and the site of entry, it seems that *L interrogans* immediately disseminate to the other organs (lung, eye, brain, liver, and kidneys) [186]. In developing and developed countries the *R norvegicus* is the major reservoir for the *Leptospira* spp. which is mostly located in urban slum areas [187]. The results of a study in the Netherlands showed that 33%–57% of *R norvegicus* isolated from 4 locations of this country were positive for *Leptospira* spp.; moreover, the results of this study indicated that in the Netherlands including areas with a poor incidence of human leptospirosis, the presence of *Leptospira* spp. in brown rats is common [188]. On the other hand, in a study in Canada among 180 samples of saliva and urine collected from *R norvegicus*, only 1 saliva sample was reported positive for *Leptospira* spp. so active shedding of *Leptospira* in the saliva is unlikely to occur [189]. In another investigation by Heusera et al., 426 Norway rats were collected, the rats mostly originated from 5 European countries and other habitats. The results of this study showed that among the collected rats, in 14.3% of them the *Leptospira* DNA was present, also, the *L interrogans* sequence type 17 was identified using the PCR-based typing technique [190]. Although there is no significant difference between patients who receive antibiotic therapy and those who are not being treated, the use of appropriate antibiotics in the initial phase of leptospirosis can reduce the symptoms and duration of illness [191], [192], [193]. Doxycycline, ampicillin, amoxicillin, and penicillin G are currently used for the treatment of this disease [182,193]. The diagnosis of *L interrogans* could be conducted by the means of dark-field or phase-contrast microscopy and also by PCR-based analysis to detect bacterial DNA in the collected samples. The culture-based techniques are not highly recommended probably due to being time-consuming and non-susceptible [194]. Nevertheless, the culture media for the isolation of *L interrogans* should contain rabbit serum, bovine serum albumin, and long-chain fatty acids, and the bacteria should be incubated at a temperature between 28 and 30 °C [182]. It is worth mentioning that the bacteria can be isolated from the CSF and blood during the first week and the second to third weeks of the disease from the urine. Another method that could bring remarkable advantages for the diagnosis of this bacteria is serologic tests like the microscopic agglutination test (MAT). Despite some suggested limitations for *L interrogans* vaccines, such as the unacceptable side effect, short-term effectiveness, and incomplete protection, vaccinations against this bacterium is used in some countries. Moreover, the variable pattern of *L interrogans* transmission may act as an obstacle against the development of a suitable and effective vaccine against this bacteria [182,186,195]. Considering the studies which have reported the widespread of *Leptospira* DNA in rats worldwide, further studies are essential to investigate the potential mechanisms which can lead to an increase in prevalence of the *Leptospira* spp. in rural habitats and evaluate its effects on the public health.

## 12 *Rickettsia* spp.

*Rickettsia* is a small pleomorphic gram-negative coccobacillus, α-proteobacteria, and obligate intracellular bacteria. This family consists of 27 species, and among them 17 species are pathogenic to humans and animals [196]. Pathogenic species are divided into 2 classic rickettsial categories; including typhus group (TG) and spotted fever group (SFG), which both are transmitted by hematophagous arthropod vectors, such as louse, flea, ticks, and mites. *Rickettsia prowazekii* and *Rickettsia typhi* are members of the TG that cause Louse-borne epidemic typhus and endemic typhus (murine typhus), respectively [196,197]. SFG contains more than 25 species, among which *Rickettsia rickettsii* is the most lethal and life-threatening pathogen causing Rocky Mountain spotted fever (RMSF) disease [198]. *Rickettsia* is transmitted through tick salivary secretion. Moreover, a flea could transmit this disease through broken skin and mucosal surfaces. Furthermore, various studies have suggested that aerosols, blood transfusion, and small rodents can cause Rickettsiosis in humans [199,200]. In a study, 420 *R norvegicus* were collected from 5 European countries and different habitats, the results showed that only 0.8% of rats were infected with *Rickettsia*, so *Rickettsia* rarely infected the *R norvegicus* populations [190]. Another research investigated the presence and performance of molecular characterization of *Rickettsia* spp., in Tick species collected in France. The results showed that the ticks collected from *R rattus* were all negative for *Rickettsia* spp. [201]. Himsworth and his colleagues reported that only 0.36% of Norway and black rats collected from an inner-city neighborhood of Vancouver were positive for *R typhi* [21]. On the other hand, Tay worked on 95 wild rats in Malaysia and reported that the DNA of *Rickettsia* was detected in the tissue homogenates of 13 (13.7%) wild rats [202]. Overall, it is suggested that *Rickettsia* can enter the human body via mucous membranes (conjunctiva), skin, and lungs. Once entering the host body, *Rickettsia* binds to the recipient's cells through an outer membrane protein (OmpA, OmpB, Sca1, and Sca2) and then penetrate the cells [203]. Before the phagolysosomal fusion and exposure to the lysosomal enzymes, this bacteria lyses the phagosome membrane and escapes into the cell cytoplasm where it can make a profit from the essential nutrients for its growth and survival [204,205]. While the members of TG proliferate in the cytoplasm of host cells until cell lysis occurs, SFG-associated bacteria exploit actin polymerization for cell-to-cell transmission and thereby infecting a high spectrum of host cells [205,206]. Noteworthy, in response to *Rickettsia* infection, host endothelial cells produce reactive oxygen species (ROS), which in turn damage the host cells by lipid peroxidation of membranes [205,207]. On the other hand, the intense and widespread reaction of immune cells (CD8 cytotoxic T-lymphocytes) removes *Rickettsia*-infected cells via inducing apoptotic cell death (instead of inducing necrosis in these cells), which itself leads to the intracellular survival of the bacteria [208,209]. At the site of entry, *Rickettsia* proliferate locally and spread through the blood and lymphatic vessels by producing an eschar [199,200]. After proliferation, *Rickettsia* increase vascular permeability and thereby induce hypovolemia, hypoperfusion and edema. Given these, interstitial pneumonia, cerebral edema, kidney injury and vasculitis in the CNS are the main causes of morbidity and mortality of rickettsial infections [200,205]. Tetracycline and doxycycline are the preferred drugs for the treatment of Rickettsioses. Diagnosis of Rickettsioses is currently based on compatible clinical symptoms, immune-histochemical detection, serologic tests and amplification of rickettsial DNA by PCR-based analysis [196,210,211]. Therefore, previous studies have shown that wild rats can act as a vector for *Rickettsia* and this should be considered in control and prevention programs for this pathogen.

## 13 *Salmonella* spp.

*Salmonella* spp. is a gram-negative bacteria, belonging to the *Enterobacteriaceae* family which can cause zoonotic infections between animals and humans [212]. *Salmonella* genus has 2 species, including *Salmonella enterica* and *Salmonella bongori* [213]*. S enterica* is the most well-known member of this family which is responsible for approximately 99% of all infections in animals and humans [214]. On the other hand, only less than 1% of human infections are caused by *S bongori,* and this species is predominantly found in cold-blooded animals [215]. This zoonotic pathogen is considered a significant public health problem, worldwide. Since this organism could grow under both anaerobic and aerobic conditions, the detection and identification of bacteria in laboratory conditions are not challenging [216]. *Salmonella* causes various infections ranging from chronic asymptomatic infections to acute febrile illness [217]. However, 3 important diseases in individuals are reported to be attributed to *Salmonella* species, which are as follows; I) typhoid fever (*Salmonella* serotype Typhi and serotype Paratyphi A, B, and C); II) invasive non-typhoidal salmonellosis; III) non-invasive non-typhoidal salmonellosis [218]. According to the WHO reports, it has been suggested that 16 to 33 million new cases of typhoid fever are diagnosed, annually, which are the cause of approximately 500,000 to 600,000 deaths worldwide [219]. Moreover, 9% of diarrheal gastrointestinal diseases are caused by *Salmonella* species worldwide [220]. Having established that the *Salmonella* genus can be transmitted in several ways, it has been suggested that contaminated water and food are the main sources of spreading this pathogen to human beings [221]. In a study conducted in Trinidad and Tobago, 204 rats were trapped and standard methods were used to isolate *Salmonella*. Only 4 rats (2.0%) out of 204 rats, were positive for *Salmonella* spp. and 75% of them were resistant to 9 antibiotics. Furthermore, this study reported that rodents can be potential carriers of multiresistant *Salmonella* spp*.* and they can affect the characteristics and epidemics of human salmonellosis although the number of recovered isolates is low [129]. Another study conducted in Thailand investigated 110 rodents, including *R norvegicus*, and *R. exulans,* which were trapped in 8 wet traditional markets. The isolates of the rodents were serotyped and were monitored for the *Salmonella*. The results indicated that 49.10% of the rodents were positive for *Salmonella*, but varied between 0% and 73.3% among markets. Moreover, having close contact with humans and food, rodents that reside in wet markets are a potential carrier of *Salmonella* [222]. Furthermore, Hilton et al. reported that among 100 fecal samples collected from the *R norvegicus* in the UK, 8% of them were positive for *S enterica* [223]. Noteworthy, in another research conducted in Canada, the result showed that *Salmonella* spp. was detected in 3 rats (0.5%) out of 633 rats with the following identified serotypes: Derby, Indiana, and Enteritidis [159]. More interestingly, it has been suggested that HIV infection, hemoglobin disorders, malignancies, and immune suppression could increase the risk of *Salmonella* infection in individuals [221,224]. According to the WHO reports in 2016, there is a difference between *Salmonella* serotypes distributed all over the world. As serotypes Typhi and Paratyphi A (typhoidal *Salmonella*) are predominantly detected in Southeast Asia, non-typhoidal *Salmonella* is more prevalent in Africa [225]. Multiple virulence factors have been enumerated for *Salmonella* infections[214]. Despite being time-consuming, conventional blood culture techniques are still considered a “gold standard” for the diagnosis of invasive salmonellosis [213]. Moreover, for the diagnosis of chronic carriers in *Salmonella typhi* infections, the use of Vi capsular antigen in the agglutination test has been suggested [214]. Furthermore, 2 pioneer methods, PCR and DNA probes are now considered an effective assay in the diagnosis of typhoid fever in recent investigations. For the therapeutic strategies, the substitution of water and electrolytes is still the main treatment suggested for *Salmonella* intestinal infections [221,226].

## 14 Conclusion

This review article sheds light on the importance of various diseases transmitted from wild rats to humans and provided a complete list of zoonotic bacterial pathogens associated with this rodent. *R norvegicus* is present in urban environments and is considered an important reservoir for various zoonotic pathogens, including bacteria, viruses, parasites, and fungi. Moreover, the results of the epidemiological studies have revealed that zoonosis pathogens, in particular zoonotic bacteria, are the main sources of new emerging illnesses. Having this in mind that to what extent some rodent-borne bacterial infections could threaten human lives, like what human beings have experienced with plague and Weil's disease, it seems that the more we know about the role of rodents in the distribution of illnesses could help us to provide a better perspective for the measures that should be done for health management and hygiene strategies. It is worth mentioning that the identification, prevention, and control of emergent zoonotic bacterial diseases in urban environments require the vast collaboration of experts, including veterinarians, urban planners, microbiologist, virologists, parasitologists, physicians, epidemiologists, and public health workers. To sum up, based on the aforementioned challenges, numerous detailed and multidisciplinary studies on rodents, such as *R norvegicus* and *R rattus* should be conducted in urban environments to better understand the occurrence of zoonosis diseases, worldwide.

## Funding

This research did not receive any specific grant from funding agencies in the public, commercial, or not-for-profit sectors.

## Author contributions

TA, AS, and SS: Conceptualization; Data curation and Writing – original draft. TA, SS, AM, and AN: Conceptualization; Methodology; Project administration; and Writing – original draft. SS, NH, and TA: Data curation; Formal analysis; Writing – original draft; and Writing – review & editing. AN, NH, and AS: Language editing. All authors read and approved the final manuscript.

## Acknowledgments

We would like to thank “The Department of Bacteriology & Virology, School of Medicine, Shiraz University of Medical Science, Shiraz, Iran” for their kind cooperation.

## Declaration of competing interest

The authors declared no potential conflicts of interest with respect to the research, authorship, and/or publication of this article.

## Data available statement

All data generated or analyzed during this study are included in this published article.

## Ethics statement

Ethics approval were waived for this study because no patients’ data were reported.

## Informed consent

Informed consent was waived for this study because no patients' data were reported.
